# *Lithocarpus orbicarpus* (Fagaceae), a new species of Stone Oak from Phang Nga province, Thailand

**DOI:** 10.3897/phytokeys.34.6429

**Published:** 2014-02-11

**Authors:** Joeri S. Strijk, Sukontip Sirimongkol, Sukid Rueangruea, Nikom Ritphet, Voradol Chamchumroon

**Affiliations:** 1 Ecological Evolution Group, Key Laboratory of Tropical Forest Ecology, Xishuangbanna Tropical Botanical Garden, Chinese Academy of Sciences, Menglun, Mengla, Yunnan, 666303, PR China; 2 Forest Herbarium (BKF), Department of National Parks, Wildlife and Plant Conservation, 61 Phahonyothin Rd., Chatuchak, Bangkok 10900, Thailand

**Keywords:** *Lithocarpus orbicarpus*, new species, Stone Oaks, Fagaceae, Thailand, Ton Pariwat Wildlife Sanctuary

## Abstract

A new species is described, *Lithocarpus orbicarpus* Strijk, collected from Ton Pariwat Wildlife Sanctuary, Mueang district, Phang Nga Province in the Peninsular Floristic Region of Thailand. We provide the first technical illustrations and colour photographs of the new species, as well as a description of its conservation status and the collecting locality. The species can be easily distinguished by its unique orbicular acorns, each covered with a dense pattern of irregularly placed scales, which completely conceal the nut, except for a tiny apical pore, and which are arranged in a dense cluster on an erect woody spike. We also provide an amendment to the existing diagnostic key to *Lithocarpus*, and discuss important differences with morphologically similar species found in Thailand and the surrounding region.

## Introduction

*Lithocarpus* Blume is the second largest genus within Fagaceae, with over 300 species described (Camus 1948, 1954; Phengklai 2008; Soepadmo 1972; Wu et al. 1999). Members of the genus, commonly called Stone Oaks, can be found in (sub-) tropical broad-leaved evergreen forests near sea level to mixed forests at altitudes of over 3200 m. The geographic distribution of *Lithocarpus* roughly covers southern and south-eastern Asia from eastern India to southern Japan, to the Philippines and southward to New Guinea. *Lithocarpus densiflorus* (Hook. & Arn.) Rehder, the only North American member of the genus was recently moved to a new monotypic genus (Manos et al. 2008). All species within *Lithocarpus* are trees, ranging from small understory elements, to very large emergent trees. Many species have a geographically wide distribution and in some locations may constitute the main canopy forming elements together with other Fagaceae (*Lithocarpus*, *Quercus*, *Castanopsis*), Lauraceae and Theaceae. Leaves are simple, entire, rarely serrate, generally glabrous and mostly spirally arranged. Male and female flowers are white to pale whitish-yellow. Genders can be either on separate inflorescences, on the same inflorescence mixed throughout, or with female flowers below and male flowers terminal, arranged with dense indumentum on erect spikes. Male flowers are solitary or in clusters of three or more, with the perianth campanulate or cup-shaped, usually 6-lobed, partially united; stamens generally 12. Female flowers solitary or in clusters of three, perianth like male flowers but smaller; 12 staminodes; 3(-4) styles (Camus 1948, 1954; Phengklai 2008).

Previous studies and the most recent treatment for Thailand have recovered 57 species of *Lithocarpus* (Barnett 1940; Phengklai 2004, 2008). During fieldwork in remote Ton Pariwat Wildlife Sanctuary, carried out as part of ongoing research on the genomics, systematics, biogeography and evolution of Asian Fagaceae, we made collections of an individual tree with unique features that could not be matched with any previously described taxa in Fagaceae. After careful examination of herbaria and literature, comparison of other specimens collected during the fieldwork and consultation of specialists on the regional flora, we report this collection here as a new species, placed within the genus *Lithocarpus*.

## Taxonomy

### Lithocarpus orbicarpus

Strijk sp. nov.

urn:lsid:ipni.org:names:77135982-1

http://species-id.net/wiki/Lithocarpus_orbicarpus

Fig. 1 , 2

#### Type.

THAILAND, Ton Pariwat Wildlife Sanctuary, Mueang district, Phang Nga Province, 8°37'25"N; 98°33'14"E; alt. 455 m, 16 July 2013, Chamchumroon et al. 5823 (Holotype: BKF; Isotypes: E, K, L, SING).

#### Diagnosis.

*Lithocarpus orbicarpus* is a small-medium sized tree. It differs from similar species by its unique orbicular acorns, each covered with a dense pattern of irregularly placed scales, which completely conceal the nut, except for a tiny apical pore, and which are arranged in a dense cluster on an erect woody spike. Unique for Thai species of *Lithocarpus*, almost the entire surface of the round nut is covered with scar area (receptacle tissue), leaving only the topmost part of the nut covered with a thin vestigial exocarp layer. Pending discovery of additional individuals, the species appears to be locally restricted to low-mid-elevation forests in the peninsular region of Thailand.

#### Description.

Small-medium sized tree, up to 15 m tall. *Bark* smooth to slightly rough grey-green, with superficial horizontal lines. Sapwood white to yellow, with inner bark ridges forming light brown longitudinal slits in sapwood surface. *Branches* dark brown to grey brown, mostly glabrous, densely lenticellate; young twigs, leaf buds and old fruits with short, soft (occasionally long) gray indumentum. Leaf buds tiny and terminal buds solitary. *Leaves* simple; lamina elliptic to oblanceolate with (strongly) acuminate tip, 11.0–22.3 × 4.4–7.1 cm. Margin entire. Leaves often with slightly asymmetric lamina. Leaf apex acuminate to strongly acuminate, leaf base cuneate to slightly attenuate. Both surfaces generally glabrous except emerging leaf buds, terminal shoots and young leaves, which have soft grey indumentum. Young leaves light green, but turning dark green above and glaucous below when older. *Venation*. Pinnately veined; secondary venation discretely anastomosing near the leaf margin. Pairs of secondary veins 9–13, slightly raised and clearly visible on underside of leaf. *Peduncles* carrying fruits 5–11 cm long, up to 1 cm thick at the base, glabrescent, grey-brown and densely lenticellate. *Male and female inflorescences* not seen. *Infructescence* a woody spike, terminal, up to 15–21 cm long. Fruits sessile on thick woody peduncle, closely pressed against each other, but walls of individual units not fused. Number of fruits per infructescence very variable, ranging from 9–20 units. *Acorn*. Orbicular, globose, 2.7–3.4 by 2.9–3.5 cm (including cupule) and covered with glabrous, semi-concentric interlocking ridges when young, which transform over time into ridges with irregularly placed scales. Cupule nearly completely enclosing the nut, indehiscent, but showing small cracks when mature; fruit wall up to 4–6 mm thick, apical pore very small, 1–4 mm wide, exposing the persistent punctiform styles (3) and a tiny fraction of vestigial exocarp. Young cupule walls light green, ridges light to dark brown. Old cupule walls turning light brown to yellow-brown and pubescent with short (occasionally long), greyish-yellow indumentum. *Nut* 1 in each cupule, ball shaped, globose, 2.4–2.9 by 2.6–3.0 cm. Up to 95% of surface area of the nut made up of scar area (receptacle tissue), upper 5% of surface area of the nut slightly raised and made up of vestigial exocarp layer. Nut scar pale yellow-whitish, tiny exocarp layer light brown. Scar area covered with deep groves and red-brown to purplish vein-like lines, stretching down to the base of the nut. Up to 5/6 of the scar area of the young nut (from the base upward) covered with dotted pattern of small depressions. Cotyledons black when dried.

**Figure 1.**
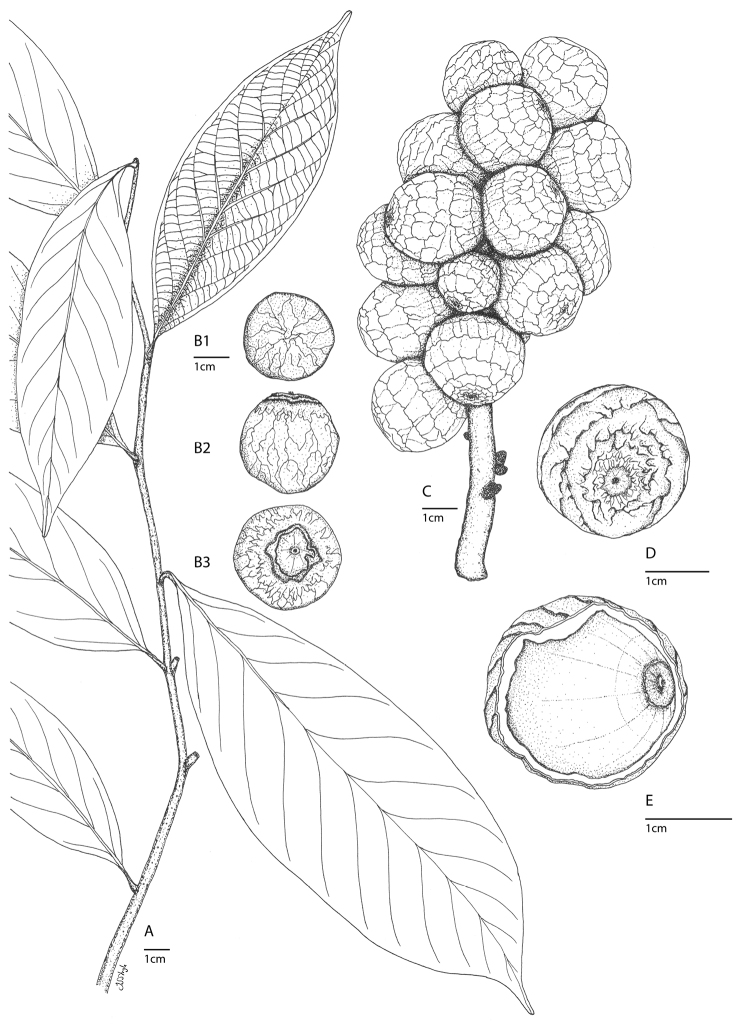
*Lithocarpus orbicarpus* Strijk, sp. nov. Chamchumroon et al. 5823 (BKF). Technical illustration. **A** Habit **B** Detail of glabrous young fruit with ridges and apical pore **C** Detail of interior of young fruit, showing nearly complete fruit scar, covered umbo and 'pitted pattern'; on the nut surface **D** Infructescence with ripe fruits showing highly irregular scaly patterns on the fruit exterior **E** Details of seed, from left to right: bottom view, side view, top view. Note venation and crevice pattern on surface of fruit, and cover of the umbo section. All drawings by J.S. Strijk.

**Figure 2.**
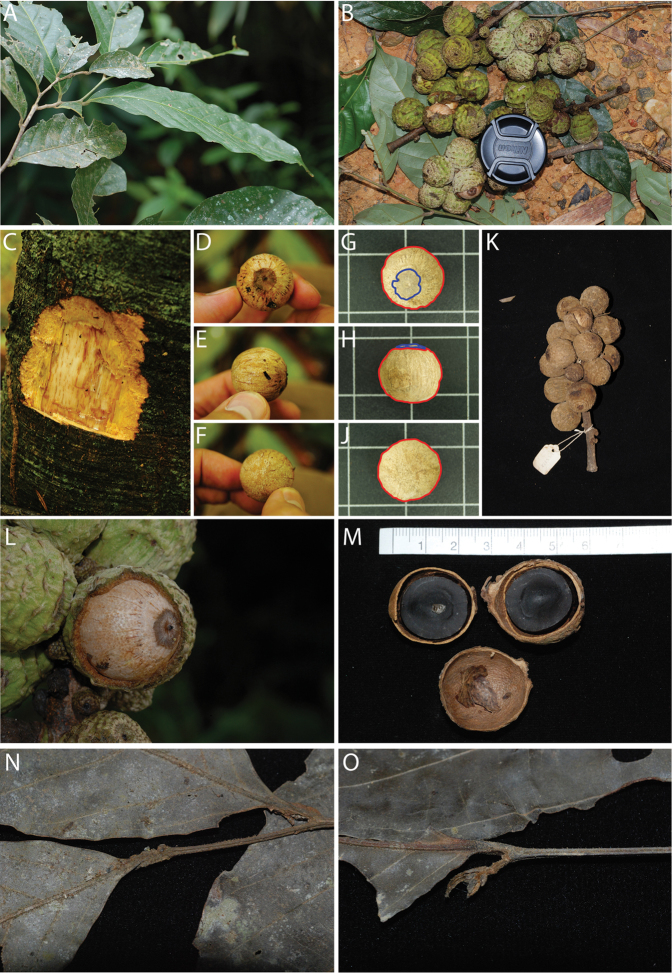
*Lithocarpus orbicarpus *Strijk, sp. nov. Chamchumroon et al. 5823 (BKF). Pictures from field collection. **A** Branch with leaves **B** Young infructescences **C** Bark and sapwood **D** Fresh fruit – top view **E** Fresh fruit – side view **F** Fresh fruit – bottom view **G** Dried fruit – top view **H** Dried fruit – side view **J** Dried fruit – bottom view (**G–J**: blue lines demarcate vestigial exocarp; red lines demarcate scar area (receptacle tissue)) **K** Dried infructescence **L** Young acorn, opened up to show dotted pattern of small depressions and surface structure of the umbo **M** Cross-section of fried nut, showing seed coat and black cotyledons **N** Terminal leaf, twig and very small terminal bud, showing grey indumentum **O** Young emerging leaf with soft grey indumentum. All pictures by S. Sirimongkol and J. S. Strijk.

#### Flowering and fruiting.

Flowering unknown, but thought to be around November-January. Infructescences on the ground in July, fruiting commencing possibly as early as April-May.

#### Distribution.

This species is only known from Thailand, and has not been recorded outside Ton Pariwat Wildlife Sanctuary, Mueang district, Phang Nga Province. During our field survey, we found only one individual tree, located on a gentle sloping section of closed dense forest. Additional survey work will have to be undertaken to determine the actual population size of this species within the wildlife sanctuary.

#### Ecology.

This species grows in dense hillside forest at an elevation of around 450 m.

#### Vernacular name.

Unknown.

#### Etymology.

*Lithocarpus orbicarpus* is named after its unique orbicular acorns, arranged densely clustered on an erect spike, of which the cup almost completely encloses the nut (except for a tiny apical pore). The exterior of the cup is covered with a dense pattern of ridges, transforming with age into horizontal and vertical lines with irregularly placed scales. Apart for a tiny portion of the umbo which is covered with vestigial exocarp, the exterior surface of the nut is completely covered by scar. Although the cupule nearly completely covers the nut, the latter is free and not fused to the wall of the cupule – a condition which occurs throughout the genus (Cannon 2001; Cannon and Manos 2001). Upon drying, the shrinking of the cotyledons inside the nut causes the whole infructescence to make a rattling sound when shaken.

#### Discussion.

Thailand has a total of 121 species, 2 subspecies and 2 varieties of Fagaceae in four genera indigenous to the country. Of these, nine species (*Castanopsis*: 4; *Quercus*: 1; *Lithocarpus*: 4) and 1 subspecies (*Quercus*: 1) are national endemics (Bunpha et al. 2011; Phengklai 2008). Three of the endemic *Lithocarpus* species (including *Lithocarpus orbicarpus*) are restricted to small ranges in the peninsular region. Of the 58 *Lithocarpus* known to occur in Thailand (including this newly described species), 43 species (74%) can be found in the Peninsular floristic province making it the most diverse floristic region (in terms of *Lithocarpus* species) followed by the North (25 species – 43%). While the Northern (and North-eastern) floristic province is characterized by species that reach high elevation habitat (>75% can be found between 1200–2500 m), the Peninsular complement includes species that are restricted to lower elevations (21 species restricted <500 m asl). With two exceptions (*Lithocarpus falconeri* (Kurz) Rehder and *Lithocarpus erythrocarpus* (Ridl.) A.Camus), none of these species are shared with the North (or North-east). In contrast, four of the Peninsular lowland species (*Lithocarpus lucidus* (Roxb.) Rehder; *Lithocarpus maingayi* (Benth.) Rehder; *Lithocarpus reinwardtii* (Korth.) A.Camus; *Lithocarpus tubulosus* (Hickel & A.Camus) A.Camus) are shared with the South-eastern floristic province, in addition to *Lithocarpus elephantum* (Hance) A.Camus and *Lithocarpus pierrei* (Hickel & A.Camus) A.Camus, which can only be found in the South-eastern region.

*Lithocarpus* in Thailand can be further characterized based on their acorn (cupule+nut) properties. The open- or semi-open type, in which the cupule shape ranges from saucer shaped-flat to covering up to 4/5th of the nut is present in 37 species (64%), while the remaining 18 species have cupules that entirely cover the nut, in most cases leaving a tiny portion of the umbo uncovered. With one exception (*Lithocarpus truncates* (King ex Hook.f.) Rehder), all closed-type species occur in the Peninsula, and eight are restricted to it (two shared with the Southeast). *Lithocarpus orbicarpus* resembles species in this group, with its indehiscent and near-closed cupules, restricted geographical distribution and presence in lower elevation habitat, but is clearly distinct from them based on the structure and shape of the fruit, the infructescence and the properties of the nut.

During our field survey, we encountered and collected additional Fagaceae species, e.g. *Lithocarpus reinwardtii* (Korth.) A.Camus (Burma, Cambodia, Malaysia, Indonesia), *Lithocarpus sundaicus* (Blume) Rehder (Malaysia, Indonesia, Brunei), *Lithocarpus cantleyanus* (King ex Hook.f.) Rehder (Burma, Malaysia, Singapore) and several, as of yet, unidentified collections. Additional species encountered in previous surveys in the wildlife sanctuary are: *Castanopsis javanica* (Blume) A.DC. (Vietnam, Malaysia, Singapore, Indonesia); *Castanopsis nephelioides* King ex Hook. f. (Malaysia, Singapore); *Castanopsis purpurea* Barnett (Thailand (endemic)); *Castanopsis wallichii* King ex Hook. f. (Malaysia, Singapore, Indonesia); *Castanopsis inermis* (Lindl.) Benth. & Hook. f. (Burma, Malaysia, Singapore, Indonesia, Philippines); *Lithocarpus bennettii* (Miq.) Rehder (Malaysia, Singapore, Indonesia); *Lithocarpus clementianus* (King) A.Camus (Malaysia, Indonesia); *Lithocarpus eucalyptifolia* (Hickel et A.Camus) A.Camus (Burma, Vietnam, Cambodia); *Lithocarpus falconeri* (Kurz) Rehder (Burma, Malaysia); *Lithocarpus garrettianus* (Craib) A.Camus (China, Burma, Laos, Vietnam); *Lithocarpus lucidus* (Roxb.) Rehder (India, Malaysia, Singapore, Indonesia, Brunei); *Lithocarpus macphailii* (Henders.) Barnett (Malaysia, Indonesia); *Lithocarpus wrayi* (King) A.Camus (Vietnam, Malaysia, Indonesia) and *Quercus oidocarpa* Korth. (Burma, Vietnam, Malaysia, Indonesia). Out of these 17 species, at least 14 have a geographic distribution that is primarily or completely located in the Sundaland biogeographic region. Conversely, only three species have a distribution that is mostly or entirely contained within the Indochinese biogeographic region. Despite the paucity of detailed biological records on this remote area, the distributional data shows us that the Fagaceae flora in Ton Pariwat Wildlife Sanctuary is distinctly Sundaic in composition, and differs substantially from forests in the more northern Indochinese region. This is in fact not surprising, as the Ton Pariwat Wildlife Sanctuary is located near the southern edge of one of the world’s major phytogeographic and zoogeographic transition zones: the ~500km stretch between the biogeographically distinct and well-known Isthmus of Kra – the narrowest part of the connection between mainland Southeast Asia and the Malay Peninsula (10°30'N) – and the line between Kangar (Malaysia) - 6.°51’N, and Pattani (Thailand) 6°87'N. Within this relatively short distance, an abrupt and major shift takes place from northern Indochinese flora and fauna, to those distinct for the southern Sundaland region (Hughes et al. 2003; Meijaard 2009; Parnell 2013; Van Steenis 1950; Woodruff 2003; Woodruff and Turner 2009).

When looking at the Fagaceae flora in the wider region, some properties of *Lithocarpus orbicarpus* resemble species in Peninsular Malaysia, such as *Castanopsis nephelioides* King ex Hook.f., but can easily be distinguished from species within *Castanopsis* (D.Don) Spach, based on the presence of bark ridges that penetrate the sapwood, the nut which is not fused to the cup and the orbicular shape of the fruit. Congeneric species from the Indo-Chinese zone that share some characters with *Lithocarpus orbicarpus* are *Lithocarpus rouletii* (Hickel & A.Camus) A.Camus (but fruit with basal scar, flattened, dehiscent; South Vietnam); *Lithocarpus pachycarpus* (Hickel & A.Camus) A.Camus (but leaves with yellow indumentum, cupules pear-shaped, acorns not orbicular; Vietnam-Laos); *Lithocarpus kontumensis* A.Camus (but cupules truncate, higher than acorn, cupules sometimes fused; Vietnam-Laos); *Lithocarpus lepidocarpus* (Hayata) Hayata (but cupules truncate, sometimes fused, fruit not orbicular; central and south Taiwan); and *Lithocarpus laoticus* (Hickel & A.Camus) A.Camus (but cupule ovoid, high elevation habitat (Tibet, south and central China, Vietnam) (Wu et al. 1999). Within Thailand, *Lithocarpus orbicarpus* is unique in its combination of properties, and we outline some of the defining differences with Thai species in Table 1 below.

This species is endemic to Thailand and is currently only known from one location in Ton Pariwat Wildlife Sanctuary. The sanctuary covers a region of low-lying forested mountains with a total area of approximately 100,000 ha at the southern end of the Phuket mountain range. As such it is an integrated part of the Southern Forest Complex of Thailand. The sanctuary is popular for its rich bird- and wildlife (e.g. Blue-banded Kingfisher (*Alcedo euryzona* Temminck, Alcedinidae) and Whitehanded Gibbons (*Hylobates lar* L., Hylobatidae) as well as rare flora, such as *Rafflesia kerrii* Meijer (Rafflesiaceae). Its unique species composition, high diversity and relatively intact forest structure underscore the importance of strengthening ongoing and future conservation measures at Ton Pariwat Wildlife Sanctuary, as a key element of wider conservation efforts in southern Thailand.

**Table 1.** Morphological differences between *Lithocarpus orbicarpus *and other Thai species of Fagaceae.

Characters	*Lithocarpus orbicarpus* Strijk	*Lithocarpus encleisocarpus* A.Camus	*Lithocarpus wrayi* (King) A.Camus	*Castanopsis nephelioides* King ex Hook.f.
1. Nut wall	Free from the cup	Free from the cup	Free from the cup	Fused to the cup
2. Cupule enclosure	Almost complete, but small apical pore showing flat umbo remains (≤5%). Indehiscent.	Almost complete, but raised umbo free (±5-10%). Easily dehiscent in irregular parts.	Almost complete, but raised umbo free (±5-15%). Indehiscent.	Enclosure complete. Indehiscent.
3. Nut shape	Orbicular.	Ovoid to globose.	Broadly conical.	Ovoid, usually depressed to one longitudinal side.
4. Cup surface	Spines absent. Small, flattened scales present. Irregularly intersecting lines present.<br/> Old acorns pubescent with short (occasionally long), greyish-yellow indumentum.	Spines and scales absent. Wall smooth, densely greenish-brown hairy.	Alternate pseudo-spines and free scales present; pseudo-spines incurved or erect.	Sparsely covered with short, woody spines, 2-3 branched reclining and decurved.
5. Acorn shape	Orbicular, symmetric; young fruits occasionally slightly skewered in young and dense infructescences	Ovoid or turbinate.	Broadly ovoid.	Obovoid, always asymmetric, usually flattened adaxially.
6. Leaf margin	Entire throughout.	Entire throughout.	Entire throughout.	Entire or serrate in the upper half.
7. Scar position, shape and size	Orbicular, covering ≥95% of the fruit, from the base upward.	Basal, slightly concave, ca. 1 cm in diameter.	Basal, concave, ca. 1.5 cm in diameter.	- (nut fused to wall).
8. Nut indumentum	Glabrous.	Greyish pubescent.	Sparsely sericeous then dull brown.	- (nut fused to wall).

## Updated key for the species of *Lithocarpus* occurring in Thailand

Following the treatment of Fagaceae for the Flora of Thailand (Phengklai 2008), no further updates have been published. In the updated key we include here, we incorporate the identification of *Lithocarpus orbicarpus* and add several additional corrections.

### Key to the thai species of *Lithocarpus*

(based on vegetative characters and acorns)

**Table:** 

1	Outer surface of cupules with annular or lamellate markings or markings lacking	
2	Cupules without lamellae, chartaceous or subcoriaceous, enclosing nearly all of the nut, more or less dehiscent when mature	
3	Cupules weakly dehiscent from the apex, cupule surface distinctly undulate with vertical and horizontal lines	
4	Cupule urn-shaped	
5	Cupule base broadly conical, much broader than apex, skin distinct with many vertical filiform lines or without. Nut conical	5. *Lithocarpus blumeanus*
5	Cupule base obconic, much narrow than apex, surface distinct with 3-4 horizontal filiform lines. Nut obconical	33. *Lithocarpus maingayi*
4	Cupule top or globe shaped	
6	Cupule top-shaped, enclosing 4/5 of nut, surface with 2–6 distinct horizontal, filiform lines	30. *Lithocarpus macphailii*
6	Cupule globe-shaped, enclosing nut completely, except for a tiny section at the apex, surface with distinct irregularly placed scales along 5–9 horizontal and vertical lines	36. *Lithocarpus orbicarpus*
3	Cupules readily dehiscent into irregular parts from the top, surface with 2–5 filiform, undulate, horizontal lines	
7	Cupules with 2 or 3 such lines	18. *Lithocarpus encleisocarpus*
7	Cupules with 4 or 5 such lines	37. *Lithocarpus pattaniensis*
2	Cupules with distinct lamellae, coriaceous, enclosing a variable amount of the nut, indehiscent	
8	Cupule enclosing not less than 1/2 of the nut	
9	Cupule enclosing about 1/2 of the nut	
10	Nuts ovoid to conical at apex, scar shallowly concave or flattened	24. *Lithocarpus gracilis*
10	Nuts subhemispheric or depressed at apex, scar deeply concave	8. *Lithocarpus clementianus*
9	Cupule enclosing not less than 3/4 of the nut	
11	Cupules obconic, enclosing nut almost completely except around the umbonate apex	
12	Nut longer than broad, ca. 1 by 0.7 cm	26. *Lithocarpus hendersonianus*
12	Nut shorter than broad, 1–2.7 by 2–3 cm	32. *Lithocarpus magnificus*
11	Cupules saucer-shaped, enclosing ca. 3/4 of the nut	1. *Lithocarpus aggregatus*
8	Cupule enclosing not more than 1/4 of the nut	
13	Nuts hemispheric or depressed on both sides	
14	Cupule enclosing 1/5 to 1/4 of the nut	39. *Lithocarpus platycarpus*
14	Cupule enclosed only the base of the nut	
15	Acorns sessile. Scar deeply concave	15. *Lithocarpus eichleri*
15	Acorns with stalk up to 0.5 cm long. Scar slightly concave	6. *Lithocarpus cantleyanus*
13	Nuts conical to broadly ovoid, or with a dome-shaped apex	
16	Cupule enclosing only the base of the nut	
17	Acorns sessile. Leaves oblanceolate	29. *Lithocarpus lucidus*
17	Acorns with fruit-stalk up to 0.5 cm long. Leaves oblong	43. *Lithocarpus reinwardtii*
16	Cupule enclosing ca. 1/4 of the nut	
18	Nut with one horizontal ring around equator. Leaves ensiform to linearlanceolate	28. *Lithocarpus loratefolius*
18	Nut without horizontal ring. Leaves ovate, ovate-oblong or narrowly elliptical	
19	Nut ovoid or conical. Cupules cup or saucer-shaped. Leaves ovate or ovate-oblong, apex caudate	3. *Lithocarpus bancanus*
19	Nut broadly ovoid. Cupules slightly obconical to saucer-shaped. Leaves narrowly elliptical	41. *Lithocarpus rassa*
1	Outer surface of cupules with alternate lamellae (resembling fish scales) or pseudospines	
20	Mature cupules of one infructescence more or less fused together	
21	Acorns broader than long, depressed both on top and at base. Cupules saucer- or cupshaped or obconic, some hardly distinct from each other through fusion	
22	Infructescences with densely arranged cupules	
23	Cupules barely distinct, resembling a large gall	13. *Lithocarpus echinophorus*
23	Cupules distinct, saucer-shaped	
24	Nut flattened or apiculate at apex, to 2.2 cm diam. Leaves cuneate at base	16. *Lithocarpus elegans*
24	Nut retuse at apex, not less than 3 cm diam. Leaves auriculate at base	2. *Lithocarpus auriculatus*
22	Infructescences with spaces between cupules	
25	Rachis of infructescence always with sub-branches. Acorns stalked	34. *Lithocarpus mekongensis*
25	Rachis of infructescence without sub-branches	
26	Acorns sessile	24. *Lithocarpus finetii*
26	Acorns stalked	50. Lithocarpus tenuinervis*
21	Acorns longer than broad, conical, ovoid or turbinate. Cupules cup-shaped or cylindric	
27	Rachis of infructescence always with sub-branches. Acorns stalked, nuts shining	
28	Acorn up to 1 cm high. Rachis up to 4 mm in diam.	7. *Lithocarpus ceriferus*
28	Acorn not less than 1 cm high (to 2.5 cm). Rachis not less than 4 mm in diam	40. *Lithocarpus polystachyus*
27	Rachis of infructescence without sub-branches. Acorns sessile, nuts more or less shining	
29	Twigs glabrous or sparsely pubescent then glabrous	
30	Cupules cup-shaped, enclosing up to 1/2 of the nut	12. *Lithocarpus dealbatus*
30	Cupules turbinate, enclosing the whole nut, open only around umbo	53. *Lithocarpus truncatus*
29	Twigs ferruginous or tomentose	
31	Leaves glabrous except along midrib. Cupules enclosing up to 1/3 of the nut	25. *Lithocarpus harmandianus*
31	Leaves densely tomentose especially on lower surface. Cupules enclosing 1/2 of the nut	27. *Lithocarpus lindleyanus*
20	Mature cupules of one infructescence, free, not fused	
32	Acorn longer than broad, conical, ovoid or obconical. Cupules cup- or saucer-shaped or obconic	
33	Cupules enclosing nut completely or 2/3 of the nut	
34	Cupules enclosing ca. 2/3 of the nut	
35	Cupules slightly obconical-shaped, nuts hairy at style apex (if persistent)	45. *Lithocarpus rufescens*
35	Cupules cup or saucer-shaped	16. *Lithocarpus elegans*
34	Cupules enclosing nut completely, or up to the apex of the nut	
36	Cupules dehiscent, obconic or ovoid	
37	Cupules obovoid, sessile, surface with dense, long and narrow recurved pseudospines	42. *Lithocarpus recurvatus*
37	Cupules ovoid, fruit stalk 2–3 mm long, surface finely ornamented with thin, triangular lamellae throughout	35. *Lithocarpus neo-robinsonii*
36	Cupules indehiscent, ovoid, surface clothed with dense, triangular lamellae	
38	Infructescences up to 18 cm long. Leaves up to 16 cm long	9. *Lithocarpus craibianus*
38	Infructescences not less than 20 cm long. Leaves not less than 20 cm long	19. *Lithocarpus erythrocarpus*
33	Cupules enclosing up to 1/2 of the nut	
39	Acorns stalked	
40	Cupules slightly obconic. Leaves ovate, ovate-oblong or obovate	48. *Lithocarpus sootepensis*
40	Cupules cup-shaped or saucer-shaped	
41	Cupules cup-shaped. Leaves lanceolate to lanceolate oblong	47. *Lithocarpus siamensis*
41	Cupules saucer-shaped to flattened. Leaves oblong to oblong-lanceolate	10. *Lithocarpus curtissii*
39	Acorns sessile	
42	Acorns (mature) not less than 3.5 by 2.2 cm	
43	Cupule lamellae bearing pseudo-spined reflexed towards the base. Leaves acute to obtuse at apex	46. *Lithocarpus scortechinii*
43	Lamellae curved towards the cupule apex. Leaves acuminate at apex	20. *Lithocarpus eucalyptifolius*
42	Acorns (mature) up to 3 by 2.2 cm	
44	Infructescence with acorns in clusters, but not fused
45	Nuts ovoid. Leaves usually curved to one side	54. *Lithocarpus wallichianus*
45	Nuts strongly apically depressed, occasionally conic. Leaves not curved	51. *Lithocarpus thomsonii*
44	Infructescence with acorns solitary, with spaces between them
46	Cupules saucer or cup-shaped, limb recurved. Leaves not less than 12 cm long	21. *Lithocarpus falconeri*
46	Cupules obconical, limb not recurved. Leaves up to 11 cm long	4. *Lithocarpus bennettii*
32	Acorns broader than long, hemisphaeric-depressed	
47	Cupules enclosing the nut completely or up to the apex of the nut	
48	Cupules more or less up to the apex of the nut, lamellae with erect or reflexed pseudospines which are not fused	
49	Pseudo-spines erect or spreading. Leaves oblanceolate. Scar nearly 1/2 of the nut	14. *Lithocarpus echinops*
49	Pseudo-spines reflexed. Leaves oblong or oblanceolate	
50	Infructescence with acorns packed close together, but not fused. Leaves slightly cuneate at base	23. *Lithocarpus garrettianus*
50	Infructescence with acorns solitary, with spaces between them. Leaves obtuse at base	54. *Lithocarpus tubulosus*
48	Cupules enclosing the nut completely, except the umbo	
51	Lamellae pointed, with narrowly pseudospines. Infructescence with acorns packed close together, but not fused	57. *Lithocarpus wrayi*
51	Lamellae flattened and imbricate. Infructescence with acorns solitary, with spaces between them	
52	Lamellae fused on lower half, the upper half free and adaxially curved	22. *Lithocarpus fenestratus*
52	Lamentas fused almost to apex, only a short free lobe adaxially curved	52. *Lithocarpus trachycarpus*
47	Cupules enclosing up to 1/2 of the nut	
53	Acorns stalked, cupules enclosing only base of the nut	
54	Stalk up to 1 cm long. Leaves glaucous on lower surface, petiole up to 1 cm long	49. *Lithocarpus sundaicus*
54	Stalk not less than 1 cm long. Leaves pale on lower surface, not glaucous, petiole not less than 1 cm long	31. *Lithocarpus magneinii*
53	Acorns sessile, cupules enclosing up to 1/2 of the nut	
55	Acorns not less than 2 by 2.5 cm	
56	Cupules slightly obconical. Leaves oblong, acute to caudate at apex, margin not revolute, petiole not less than 1 cm	11. *Lithocarpus cyclophorus*
56	Cupules saucer-shaped. Leaves obovate, obtuse at apex, margin revolute, petiole up to 0.6 cm long	44. *Lithocarpus revolutus*
55	Acorns up to 1.5 by 2 cm	
57	Nuts convex at the apex	
58	Cupules saucer-shaped to flattened and discoid. Leaves not whorled	
59	Lamellae usually fused throughout. Leaves up to 15 cm long	38. *Lithocarpus pierrei*
59	Lamellae fused at base only, apices free. Leaves not less than 18 cm long	17. *Lithocarpus elephantum*
58	Cupules cup-shaped. Leaves usually whorled at the twig tips	58. *Lithocarpus xylocarpus*
57	Nuts flattened at the apex. Cupule cup-shaped, enclosing 1/5 to 1/2 of the nut. Leaves with unequal sides, usually curved to one side	
60	Leaves oblong, elliptic oblong, not less than 10 by 3.5 cm, with 14–20 pairs of lateral nerves	55. *Lithocarpus vestitus*

## Supplementary Material

XML Treatment for Lithocarpus orbicarpus
